# An ex-ante cost-utility analysis of the deemed consent legislation compared to expressed consent for kidney transplantations in Nova Scotia

**DOI:** 10.1186/s12962-022-00390-z

**Published:** 2022-10-06

**Authors:** Prosper Koto, Karthik Tennankore, Amanda Vinson, Kristina Krmpotic, Matthew J. Weiss, Chris Theriault, Stephen Beed

**Affiliations:** 1 Research Methods Unit, Nova Scotia Health, 5790 University Avenue, Halifax, NS B3H 1V7 Canada; 2 Department of Medicine (Division of Nephrology), Dalhousie University, Halifax, NS Canada; 3 Department of Critical Care, Dalhousie University, Halifax, NS Canada; 4 Centre Mère-Enfant Soleil du CHU de Québec, Transplant Québec, Québec, QC Canada; 5 Department of Critical Care, Department of Anesthesia, Pain Management & Perioperative Medicine, Dalhousie University, Halifax, NS Canada

**Keywords:** Deceased-donor kidney transplantation, Deemed consent, Cost-utility, Ex-ante, Markov model

## Abstract

**Background:**

This study was an ex-ante cost-utility analysis of deemed consent legislation for deceased organ donation in Nova Scotia, a province in Canada. The legislation became effective in January 2021. The study's objective was to assess the conditions necessary for the legislation change’s cost-effectiveness compared to expressed consent, focusing on kidney transplantation (KT).

**Method:**

We performed a cost-utility analysis using a Markov model with a lifetime horizon. The study was from a Canadian payer perspective. The target population was patients with end-stage kidney disease (ESKD) in Atlantic Canada waitlisted for KT. The intervention was the deemed consent and accompanying health system transformations. Expressed consent (before the change) was the comparator. We simulated the minimum required increase in deceased donor KT per year for the cost-effectiveness of the deemed consent. We also evaluated how changes in dialysis and maintenance immunosuppressant drug costs and living donor KT per year impacted cost-effectiveness in sensitivity analyses.

**Results:**

The expected lifetime cost of an ESKD patient ranged from $177,663 to $553,897. In the deemed consent environment, the expected lifetime cost per patient depended on the percentage increases in the proportion of ESKD patients on the waitlist getting a KT in a year. The incremental cost-utility ratio (ICUR) increased with deceased donor KT per year. Cost-effectiveness of deemed consent compared to expressed consent required a minimum of a 1% increase in deceased donor KT per year. A 1% increase was associated with an ICUR of $32,629 per QALY (95% CI: − $64,279, $232,488) with a 81% probability of being cost-effective if the willingness-to-pay (WTP) was $61,466. Increases in dialysis and post-KT maintenance immunosuppressant drug costs above a threshold impacted value for money. The threshold for immunosuppressant drug costs also depended on the percent increases in deceased donor KT probability and the WTP threshold.

**Conclusions:**

The deemed consent legislation in NS for deceased organ donation and the accompanying health system transformations are cost-effective to the extent that they are anticipated to contribute to more deceased donor KTs than before, and even a small increase in the proportion of waitlist patients receiving a deceased donor KT than before the change represents value for money.

**Supplementary Information:**

The online version contains supplementary material available at 10.1186/s12962-022-00390-z.

## Introduction

Nova Scotia (NS), a province in Canada, enacted the Human Organ and Tissue Donation Act (HOTDA, Bill 133), which includes deemed consent legislation for deceased organ donation. Under deemed consent, eligible adults are presumed to have consented to deceased organ donation unless registered otherwise. The Bill received Royal Assent on April 12, 2019, and became effective on January 18, 2021 [1]. HOTDA includes moving from an expressed to a deemed consent environment, accompanied by health system transformations. The expectation was that the health system transformations would optimize donor yield. The HOTDA in NS is a soft deemed consent because substitute decision-makers (as defined by HOTDA) can give or withhold their consent for donation on behalf of the deceased [1, 2].

The options for increasing organ donors include expanding the donor pool and optimizing the number of organs recovered per donor [3]. Initiatives to promote organ donation, including legal frameworks within the context of transplant systems and consent mechanisms, are essential to expanding the potential donor pool. Deemed (also known as opt-out or presumed) consent and expressed (also known as opt-in) consent models are the most frequently used transplant systems [4]. A deemed consent system considers all eligible individuals—generally defined as competent adults—deceased organ donors unless they express their preference against deceased organ donation while alive [4, 5]. On the other hand, the expressed consent system requires eligible individuals or their surrogates to express their preference for deceased organ donation [4, 5]. Hard consent, soft consent, and mandated choice constitute variations of these consent mechanisms [4]. A hard consent emphasizes respect for the individual's stated preference, with no recognized role for family or substitute decision-makers, while soft consent models allow for modification of registered consent by surrogates [4]. A mandated choice as a consent mechanism requires registration of intent to or not to donate by eligible individuals [4, 5].

Since the default is presumed consent for donation in a deemed consent model, a hard deemed consent requires individuals to state their preference against deceased organ donation to avoid post-mortem organ recovery [4]. In a hard deemed consent model, donations could occur without substitute decision-makers recognized role in the absence of documentation about the deceased's wishes [4, 5]. In contrast, in a soft consent environment, there is a defined role for families or substitute decision-makers, as defined by law, irrespective of the transplant system [4]. England, Wales and Spain have a soft deemed consent policy [6], and this policy was chosen for Nova Scotia's legislative reform [1, 2].

In addition to the legislation change, and as part of the health system transformations, the NS provincial government, in 2019, committed to providing resources to fund initiatives identified by the organ donation program [7] and the critical elements of high-performing deceased donation systems as defined in the most recent system progress report of organ donation and transplantation in Canada, under Canadian Blood Services [8]. The initiates included recruiting specialist donation physicians and donor coordinators, providing professional education for frontline healthcare providers, a deemed consent registry (a centralized database), support for the Regional Tissue Bank and the Multi-Organ Transplant Program (MOTP) and public awareness campaigns [7].

According to a 2020 report by the Canadian Institute for Health Information (CIHI), at the end of 2019, there were 3084 solid organs transplanted in Canada (including Quebec) [9]. During the same period, there were 4352 persons on various waiting lists for organ transplantation (kidney, liver, heart, lung and pancreas), including 249 persons who died while waiting for transplantation. The deceased donor rate was 21.8 donors per million, and the living donor rate was 16.3 donors per million [9]. Similarly, at the end of 2019, 40,734 Canadians (excluding Quebec) lived with end-stage kidney disease (ESKD), with 56.8% on dialysis and 43.2% with functioning kidney transplantation (KT) [9]. In Canada, ESKD patients who receive a KT spend 3.7 years on dialysis before KT [9].

Queen Elizabeth II’s (QEII) Multi-Organ Transplant Program (MOTP) of Atlantic Canada, located in Halifax, NS, provides organ transplantation services for NS, New Brunswick (NB), Prince Edward Island (PEI), and Newfoundland and Labrador (NL) residents. The MOTP serves approximately 2.4 million people and maintains an organ transplantation waitlist. According to various MOTP reports, from 2014 to 2018, the QEII completed 239 transplantations, comprised of 67% kidney, 25% liver, 8% heart, and 1% pancreas. In Atlantic Canada, the mean wait time for KT was three years [9]. On Dec 31, 2020, patients with ESKD made up 87% of the 206 individuals on the transplant waiting list in Atlantic Canada [10].

The impact of deemed consent and the health system transformations on consent rates, deceased donation rates, the number of patients getting transplantations in a year, and the overall economic impact remains unknown. Therefore, part of understanding the anticipated effectiveness of the legislation was to examine the conditions required for its cost-effectiveness. In the case of KT, one deceased donor could potentially provide two kidneys, thereby doubling the value for each available donor. In addition, we expect that the deemed consent will increase consent rates, thereby increasing potential and actual donors. However, evidence from other jurisdictions suggests a mixed impact on overall donation rates. Arshad et al. compared organ donation and transplantation rates for 35 countries in the Organization for Economic Co-operation and Development (OECD)[11]. They reported that compared to countries with expressed consent, those with deemed consent had no significant difference in deceased donor and solid organ transplantation rates and had fewer living donors per million [11]. However, in a systematic review, Ahmad et al. reported that countries with deemed consent had higher deceased donation and deceased transplantation rates than those with other models [5]. The lack of conclusive evidence may be partly because experiences from elsewhere show that deemed consent was typically accompanied by other changes in the health system, making it difficult to isolate the independent impact of a deemed consent model on consent and donation rates [12, 13].

A recent systematic review of various policies to increase KT found such policies to be cost-effective [14]. The policies included payment to living donors and transplanting organs from high-risk donors, defined as donors with risk of HIV and Hepatitis C virus-infected donor kidneys, given the availability of direct-acting antiviral (DAA) therapy for hepatitis C virus and human leukocyte antigens (HLA) mismatched kidneys [14]. However, whether deemed consent in NS will be cost-effective and under what conditions remains unclear. The objective of the current study was to assess the conditions required for the deemed consent model and the accompanying health system transformations (the intervention) to be cost-effective compared to expressed consent. Deemed consent replaced expressed consent model. The current paper presents an ex-ante cost-utility analysis from a Canadian payer perspective. It was an ex-ante in that the study examined the conditions for cost-effectiveness, including setting out the threshold increases for deceased donor KT required.

## Methods

### Study design and target population

We developed a Markov model to simulate the minimum required increase in deceased donor KT per year for the deemed consent model to be cost-effective. We followed the Consolidated Health Economic Evaluation Reporting Standards (CHEERS) for analytic decision models [15]. The target population was ESKD patients on the MOTP waitlist in Atlantic Canada, 18 years and older. The legislation change occurred in NS. However, the benefits extend to other waitlisted patients in the MOTP from the other provinces in Atlantic Canada, acknowledging that NS is the transplantation center for all KT in Atlantic Canada.

### Health outcomes

The health outcomes were quality-adjusted life-years (QALYs) and health care costs. We sourced health utility scores by health states required to compute QALYs from the literature (Table 1) [16]. The expectation was that KT recipients would experience a survival advantage with higher health utilities and thus higher QALYs.

**Table 1:** Health utility and cost parameters

Parameter	Mean	SD	Distribution in PSA	Source
Annual cost of facility-based HD	$66,947	$6,695	Gamma	Beaudry et al.
Training costs for patients using PD	$7,462	$746	Gamma	Beaudry et al.
Annual cost of PD	$40,303	$4,030	Gamma	Beaudry et al.
One-time cost of organ procurement	$26,943	–	–	IHIACC
One-time cost of KT	$55,997	$4,883	Gamma	NSH
Annual cost of immunosuppressants				
Organ from living donor:				
Year one	$15,225	$1,522	Gamma	Ferguson et al
After year one	$1,953	$195	Gamma	Ferguson et al.
Organ from a deceased donor				
Year one	$14,405	$1,441	Gamma	Ferguson et al.
After year one	$2,174	$217	Gamma	Ferguson et al.
Annual inpatient costs before KT				
Organ from living donor	$24,744	$18,812	Gamma	Koto et al.
Organ from a deceased donor	$23,297	$22,655	Gamma	Koto et al.
Annual inpatient costs after KT				
Organ from living donor	$24,632	$19,200	Gamma	Koto et al.
Organ from a deceased donor	$26,291	$39,960	Gamma	Koto et al.
Annual insured physician services before KT				
Organ from living donor	$3,460	$4,527	Gamma	Koto et al.
Organ from a deceased donor	$4,905	$5,873	Gamma	Koto et al.
Insured physician services in KT year	$7,672	$5,893	Gamma	Koto et al.
Annual insured physician services after KT				
Organ from living donor	$3,373	$4,338	Gamma	Koto et al.
Organ from a deceased donor	$3,880	$3,701	Gamma	Koto et al.
Health utility for dialysis patients	0.639	0.064	Beta	CADTH
Health utility for KT recipients	0.816	0.082	Beta	CADTH

*SD* standard deviation, *IHACC* Interprovincial Health Insurance Agreements Coordinating Committee, *NSH* Nova Scotia Health*, PD* peritoneal dialysis, *HD* hemodialysis, *KT* Kidney transplantation, *CIHI* Canadian Institute for Health Information, *CADTH* Canadian Agency for Drugs and Technologies in Health, *PSA* probabilistic sensitivity analysis

### Measurement of effectiveness

We expected deemed consent to increase consent rates exceeding those opting out, thereby increasing the pool of potential and actual donors and the probability of receiving deceased donor KT for all ESKD patients on the waitlist. In practical terms, the simulated treatment effect was that the proportion of patients on the waitlist getting a deceased donor KT in a year, the probability of a deceased donor KT, will be higher in the deemed consent arm of the model than in the expressed consent arm. Our approach to modelling the effectiveness of the intervention followed the literature [3, 17]. While DeRoos et al*.* [3] simulated deceased donor increases, the current study simulated increases in the proportion of ESKD patients on the MOTP waitlist getting a deceased KT in a year following deemed consent. We evaluated a decrease of 10% to a 100% increase (-10% to 100%), using mean KTs performed from 2010 to 2018 as the baseline. Background factors such as per capita health expenditure, real per capita gross domestic product, religion, and mortality rates from motor vehicle and cerebrovascular accidents can affect organ donation rates [2, 18]; however, we assumed these factors would not deviate from long-term trends.

### Resources and costs

Costs included dialysis, one-time organ procurement, a one-time KT cost, annual immunosuppressant drug costs, annual inpatient costs before and after KT, and costs for insured physician services before, during and after KT (Table 1). In-centre hospital hemodialysis (HD) costs served as a proxy for HD costs (Table 1). We assumed that patients received dialysis thrice a week. Dialysis costs came from a Canadian study [19]. Dialysis-related costs included facility-based HD, the yearly cost of peritoneal dialysis (PD) and a one-time cost associated with training patients to use PD [19]. Dialysis costs also included direct expenses related to human resources–registered nurses, licensed practical nurses, unit clerks, dieticians, dialysis technicians, clinical pharmacists, and social workers. These direct costs included benefits, vacation and relief. In addition, the dialysis costs included medical, surgical, laboratory, housekeeping, and maintenance supplies [19]. The costs included drug, equipment, departmental sundry, overhead, water, capital, and in-center runs. The original values in Beaudry et al*.* [19] were in 2016 dollars which we converted into 2019 dollars using the Canadian consumer price index (CPI) for health and personal care.

We included a one-time organ procurement cost per patient: the in-country organ procurement cost from the interprovincial billing rates for designated high-cost transplantations of the Interprovincial Health Insurance Agreements Coordinating Committee (IHIACC) in Canada, April 2019 [20]. According to IHIACC, the organ procurement cost reflected the health resources used in procuring, storing, shipping, and maintaining the donor [20]. As a result, we assumed the same organ procurement costs for recipients of kidneys from deceased and living donors.

The one-time cost of KT came from the NS health case costing centre. The resource use units included allied health, anesthesia, electrocardiogram/echo, emergency, food services, intensive and critical care, inpatient unit, lab, medical imaging, operating room, in-hospital medications, and recovery. Direct costs included direct variable labour, drugs, traceable, variable supplies, contracted out services, fixed labour, fixed labour medical, fixed costs associated with buildings and equipment, fixed sundry, and administrative recall. These costs also included overhead and costs associated with monitoring, observation, and dialysis for those experiencing delayed graft function. Cost of KT data covered fiscal years 2015/2016, 2016/2017, and 2017/2018, with the mean for the three years included in the model after adjusting for inflation.

KT patients receive prophylaxis to reduce infectious complications during KT. These include anti-pneumocystis jirovecii pneumonia, anti-herpes simplex viruses, and anti-urinary tract infection. High-risk patients also receive anti-cytomegalovirus. In addition, patients receive induction therapy at KT. Those with a high panel-reactive antibody receive anti-thymocyte globulin, while those at a lower risk receive Basiliximab. KT patients also require maintenance immunosuppressive medications available in various combination regimens over time. We sourced medication costs from a Canadian study [21]. The medication costs varied by whether the patient received an organ from a deceased or a living donor [21]. The medication costs were higher in the first year of KT and reduced in subsequent years (Table 1).

The annual inpatient costs before and after KT and costs for insured physician services before and after KT varied by whether the patient received an organ from a deceased or a living donor (Table 1). We sourced these costs from a Canadian study [22].

The Canada Health Act governs the Canadian health insurance system, a publicly funded health care system [23]. The public sector's share of total health expenditure was 70% compared to 30% for the private sector in 2019 [24]. The public sector's share for countries in the OECD for 2019 was 73% [24]. Also, the public–private split for the United States was 49% public and 51% private in 2019. The Act mandates universal coverage for medically necessary health care and access to hospital and physician services for all insured residents based on need and not the ability and willingness to pay [23]. Consequently, the health care costs reported in this study approximate the actual costs rather than prices or charges.

At the provincial level, the provincial health insurance scheme, shaped by the Canada Health Act, covers all KT for all NS patients with a valid NS health card. Hospital visits and in-hospital drugs are publicly insured. In addition, NS operates two drug insurance programs for out-of-hospital drug costs; the Seniors' Pharmacare for NS patients 65 years and older without private coverage or coverage under any other program and the Family Pharmacare Program for all NS residents without drug coverage or residents facing high drug costs. The Family Pharmacare Program covers the cost of post-transplantation medications. All costs were in 2019 Canadian dollars.

### Model

**Fig. 1:**
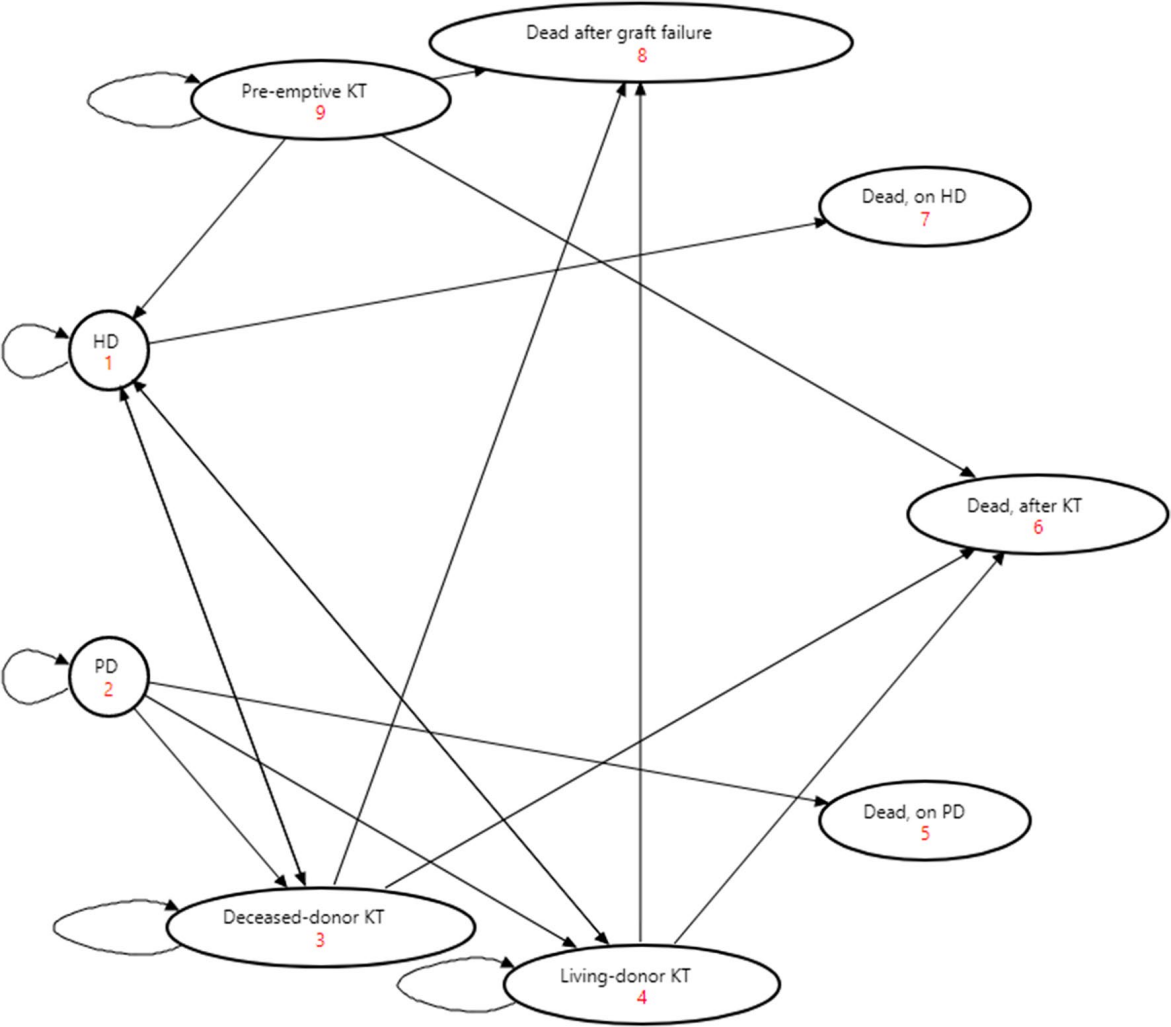
The Markov model. *KT* kidney transplantation, *HD* hemodialysis, *PD* peritoneal dialysis

We developed a Markov model as the vehicle for the cost-utility analysis (Fig. 1) [15, 25]. Markov models are helpful when a decision problem involves ongoing risk over time, when the timing of events is essential, and when events may recur [25]. In addition, they help model decision problems where patients are in specific health states [15, 25–27]. The Markov model provided the opportunity to examine patients in discrete health states and the transition between health states over a long-term horizon. The model also allowed us to model repetitive events and the time dependencies of the transitions from one health state to another. We assessed the model's validity following established guidelines [28–30]. We discounted relevant costs and QALYs at 1.5% per annum, following the Canadian Agency for Drugs and Technologies in Healthʼs (CADTHʼs) recommendations [31].

The mean age of KT recipients in the MOTP database from 2010 to 2018 was 50 years. Hence, the model assumed a cohort with a starting age of 50 years and adopted a lifetime horizon, with a cycle length of one year. There were five health states and four absorbing states in the model: pre-emptive KT, alive after receiving KT from a deceased donor, alive after receiving KT from a live donor, alive on HD, and alive on PD. The absorbing states were dead on HD, PD, KT with a functioning graft and after graft failure. After activation on the waitlist, a patient with ESKD not yet on dialysis was added to the pool of potential KT recipients and may get pre-emptive KT. A patient on dialysis may die or be removed from the waitlist without getting KT. We varied the probability of death on dialysis by the type and length of time on dialysis. The likelihood of receiving a deceased donor KT, either an organ from a donor after cardiac death (DCD) or a donor after brain (neurologic) death (DBD), was time-dependent while on the waitlist (Table 2). After a KT, a patient may experience graft failure and die or be back on dialysis and may get a re-transplantation. The probability of death after graft failure came from Kabani et al. [32]. A patient may eventually die after living with a functioning graft. Patients with pre-emptive KT had a reduced risk of graft failure and death [33].

**Table 2:** Annual transition probabilities

Probability	Mean	SD	Distribution in PSA	Source
Graft failure: KT from a deceased donor	Years 1 − 3: 0.063	0.006	Beta	Canadian Organ Replacement Register
	Years 4 − 5: 0.113	0.011	Beta	
	Years 6 − 9: 0.168	0.017	Beta	
	Years 10 + : 0.318	0.032	Beta	
Graft failure: KT from a living donor	Years 1 − 3: 0.028	0.003	Beta	Canadian Organ Replacement Register
	Years 4 − 5: 0.058	0.006	Beta	
	Years 6 − 9: 0.095	0.009	Beta	
	Years 10 + : 0.226	0.023	Beta	
Deceased donor KT	Year 1: 0.156	0.016	Beta	Estimated^a^
	Year 2: 0.230	0.023	Beta	
	Year 3: 0.278	0.028	Beta	
	Year 4: 0.314	0.031	Beta	
	Year 5: 0.335	0.034	Beta	
	Year 6: 0.364	0.036	Beta	
	Year 7: 0.378	0.038	Beta	
	Year 8: 0.402	0.040	Beta	
	Year 9: 0.411	0.041	Beta	
	Years 10 + : 0.441	0.044	Beta	
Death on hemodialysis	Years 1 − 3: 0.178	0.018	Beta	Canadian Organ Replacement Register
	Years 4 − 5: 0.335	0.034	Beta	
	Years 6 − 9: 0.446	0.045	Beta	
	Years 10 + : 0.581	0.058	Beta	
Death on peritoneal dialysis	Years 1 − 3: 0.075	0.008	Beta	Canadian Organ Replacement Register
	Years 4 − 5: 0.248	0.025	Beta	
	Years 6 − 9: 0.380	0.038	Beta	
	Years 10 + : 0.556	0.056	Beta	
Death after graft failure	Year 1: 0.121	0.012	Beta	Kabani et al.
	Year 2: 0.060	0.006	Beta	
	Year 3 + : 0.05	0.005	Beta	
Death after KT	Year 1: 0.036	0.004	Beta	Canadian Organ Replacement Register
	Year 2: 0.058	0.006	Beta	
	Year 3: 0.076	0.008	Beta	
	Year 4: 0.099	0.009	Beta	
	Years 5 + : 0.121	0.012	Beta	

Probability	Mean	SD	Distribution in PSA	Source
Pre-emptive KT	0.024	0.002	Beta	Estimated^a^
Hemodialysis	0.656	0.066	Beta	Estimated^a^
Peritoneal dialysis	0.125	0.013	Beta	Estimated^a^
Living donor KT	0.19	0.019	Beta	Estimated^a^
Reduced risk of death: pre-emptive KT	0.69	0.069	lognormal	Kabani et al.
Reduced risk of graft failure: pre-emptive KT	0.73	0.073	lognormal	Kabani et al.

^a^Authors’ estimate based on data from the Multi-Organ transplant program (MOTP)

*SD* standard deviation, *KT* kidney transplantation, *PSA* probabilistic sensitivity analysis

We assessed cost-effectiveness using the incremental cost-utility ratio (ICUR) [26]:

$$ICUR=\frac{\Delta C}{\Delta E}=\frac{{Cost}_{deemed \,\,consent}-{Cost}_{expressed\, \,consent}}{{QALYs}_{deemed \,\,consent}-{QALYs}_{expressed \,\,consent}}<\lambda$$

where λ represents the willingness-to-pay (WTP) threshold; ∆E the incremental QALYs; ∆C the incremental cost. The incremental cost equals the difference in expected costs between the model's deemed and expressed consent arms. The inputs for the incremental costs and QALYs came from the model. Canada does not have an explicit WTP threshold. Consequently, we followed the World Health Organization's (WHO's) framework in selecting the WTP threshold, using Canada's real (inflation-adjusted) gross domestic product (GDP) per capita as the WTP threshold [34]. The GDP per capita in purchasing power parity (PPP) terms (international $) for Canada in 2019 was $50,661 [35]. The PPP conversion between the US (USD) and the Canadian dollar (CAD) for 2019 was 1 USD equals 1.213 CAD [36]. Therefore, we used the conversion factor to convert the amount into Canadian dollars to derive $61,466 as the WTP threshold.

The following transition probabilities were time-dependent: the probability of getting a deceased donor KT; dead after KT with a functioning graft; graft failure (varies by the source of the organ); death after graft failure and death while on dialysis (varies by dialysis type). The time-dependent transition probabilities reflect the likelihood of moving from one state to another as the cohort ages (Table 2) [26]. The time-dependent probability of getting a deceased-donor KT came from an analysis based on the MOTP data following the literature [37]. The simulated treatment effect was that the deceased-donor KT probability was higher in the deemed consent arm of the model than in the expressed consent arm. We performed a threshold analysis to determine the minimum increase required for cost-effectiveness. The analysis involved half-cycle corrections [27]. We conducted the analysis using TreeAge Pro Healthcare 2021 R1 [38].

### Sensitivity analyses

We performed one-way and probabilistic sensitivity analyses (PSA). We varied the annual costs of HD, PD, and maintenance immunosuppressant drugs in one-way sensitivity analyses. We evaluated their impact on ICUR and reported the results using a tornado diagram. We also varied the WTP threshold from $0 to $100,000 and reported its effect on the cost-effectiveness probability. We repeated the analysis for selected changes in the proportion of patients on the waitlist getting a deceased-donor KT in a year. We also limited the time horizon to five years to examine how changes in the time horizon could impact the ICUR. In a further sensitivity analysis, we evaluated how changes in living donor KTs could impact the cost-effectiveness of deemed consent by evaluating two cases. In the first case, we assumed a 1% increase in deceased donor KT and evaluated how a 0 to 100% decrease in living donor KT affects cost-effectiveness. We assumed a 26% increase in decreased donor KT and a 0 to 100% decrease in living donor KT in the second case.

We used PSA to quantify the uncertainty around estimates. We modelled cost parameters' uncertainty using a gamma distribution, health utilities and transition probabilities, including time-dependent probabilities, using a beta distribution and lognormal distribution for reduced risks [26, 39, 40] (Tables 1 & 2). The PSA involved 10,000 Monte Carlo simulations [26, 41]. We summarized the results from the PSAs on cost-effectiveness acceptability curves (CEAC) and cost-effectiveness planes [26]. The CEAC outlines the proportion of the distribution of the incremental costs and QALYs (∆C and ∆E) that fell within the acceptable region of the cost-effectiveness plane as the WTP threshold changes [26]. We also used cost-effectiveness planes to show the uncertainty around ICUR estimates. The 95% confidence interval around the estimates came from the PSAs.

## Results

Without deemed consent, an ESKD patient's expected lifetime cost ranges from $177,663 to $553,897 (Table 3). However, at five years instead of a lifetime horizon, the cost ranged from $113,367 to $336,276 per patient (Table 3). In the deemed consent environment, the expected cost per patient depends on the percentage increases in the proportion of ESKD patients on the waitlist getting a KT in a year. A 1% increase was approximately equal to the expected costs for the expressed consent arm of the model (Table 3). However, for a 26% increase, the expected lifetime costs ranged from $177,541 to $608,737 and $116,974 to $324,983 for 5 years (Table 3). The higher expected total lifetime costs for the deemed consent arm reflected the increased probability of getting a deceased donor KT, incurring the costs associated with KT and the annual costs of maintenance immunosuppressant drugs for an extended period. The deemed consent arm had higher expected lifetime QALYs than the expressed consent arm. The lifetime QALYs in expressed consent ranged from 2.92 to 4.54 and 1.72 to 2.48 over five years. The expected QALYs in deemed consent depended on the percent increase in deceased-donor KTs and the time horizon (Table 3). Over a lifetime, a 1% increase generated QALYs from 2.92 to 4.55, while a 26% increase generated 3.06 to 4.92 QALYs (Table 3).

**Table 3:** Cost-effectiveness analysis results comparing deemed to expressed consent in deceased donor KT

Strategy	Expected Cost (95% CI)	Expected QALYs (95% CI)	∆C	∆QALYs	ICUR ($/QALY)
1% increase in deceased donor KT evaluated over a lifetime					
Deemed consent	$308,516 ($177,627 to $555,362)	3.86 (2.92 to 4.55)	$349	0.01	32,629 (95% CI: − 64,279 to 232,488) Probability of cost-effectiveness if WTP is 61,466: 81%
Expressed consent	$308,167 ($177,663 to $553,897)	3.85 (2.92 to 4.54)			
1% increase in deceased donor KT evaluated over five years					
Deemed consent	$192,453 ($113,063 to $336,939)	2.16 (1.72 to 2.48)	− $319	0.01	Expressed consent dominated
Expressed consent	$192,772 ($113,367 to 336,276)	2.15 (1.72 to 2.48)			
26% increase in deceased donor KT evaluated over a lifetime					
Deemed consent	$319,824 ($177,541 to $608,737)	4.15 (3.06 to 4.92)	$11,657	0.30	38,594 (95% CI: − 41,022 to 220,930) Probability of cost-effectiveness if WTP is 61,466: 80%
Expressed consent	$308,167 ($177,663 to $553,897)	3.85 (2.92 to 4.54)			
26% increase in deceased donor KT evaluated over 5 years					
Deemed consent	$185,057 ($108,603 to $331,539)	2.20 (1.69 to 2.56)	− $7,715	0.05	Expressed consent dominated
Expressed consent	$192,772 ($116,974 to 324,983)	2.15 (1.69 to 2.50)			

*QALYs* quality-adjusted life years, *∆C* incremental cost, ∆*QALYs* incremental QALYs, *KT* kidney transplantation, *QALYs* quality-adjusted life years, *ICUR* incremental cost-utility ratio.

95% CI from probabilistic sensitivity analysis

**Fig. 2:**
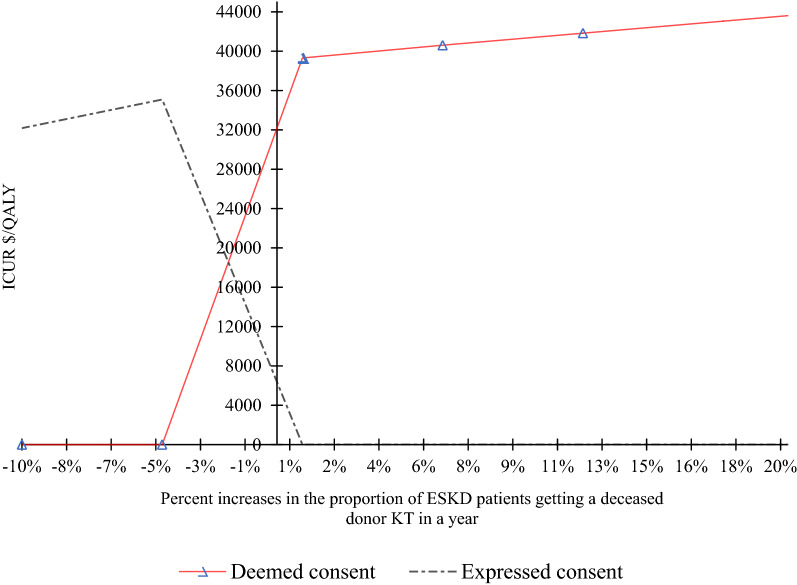
Responsiveness of ICUR to changes in the proportion of deceased donor kidney transplantations per year. *ICUR* incremental cost-utility ratio, *ESKD* end-stage kidney disease, *KT* kidney transplantation, *QALY* quality-adjusted life-year

The ICUR increased with deceased donor KTs per year (Fig. 2). For a 10% decrease, the deemed consent arm had a lifetime cost of $305,780 compared to $308,167 in the expressed consent environment, with a cost difference of $2,386. Expressed consent also had higher QALYs (3.85 versus 3.76) with a QALY difference of 0.1 (Fig. 2 and Additional file 1: Table S1).

**Fig. 3:**
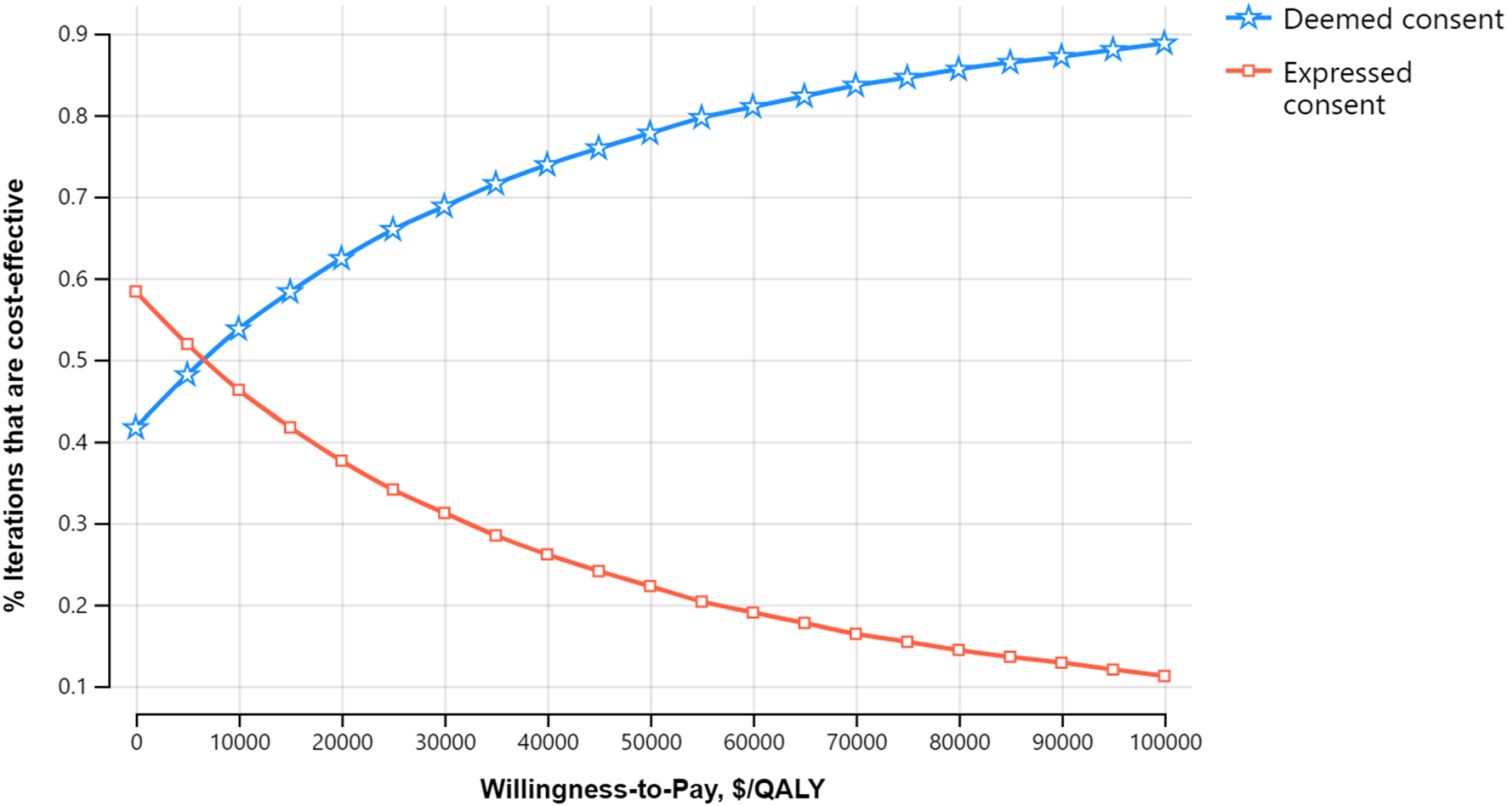
Cost-effectiveness acceptability curve for a 1% increase in deceased donor kidney transplantation probability. *QALY* quality-adjusted life-year

**Fig. 4:**
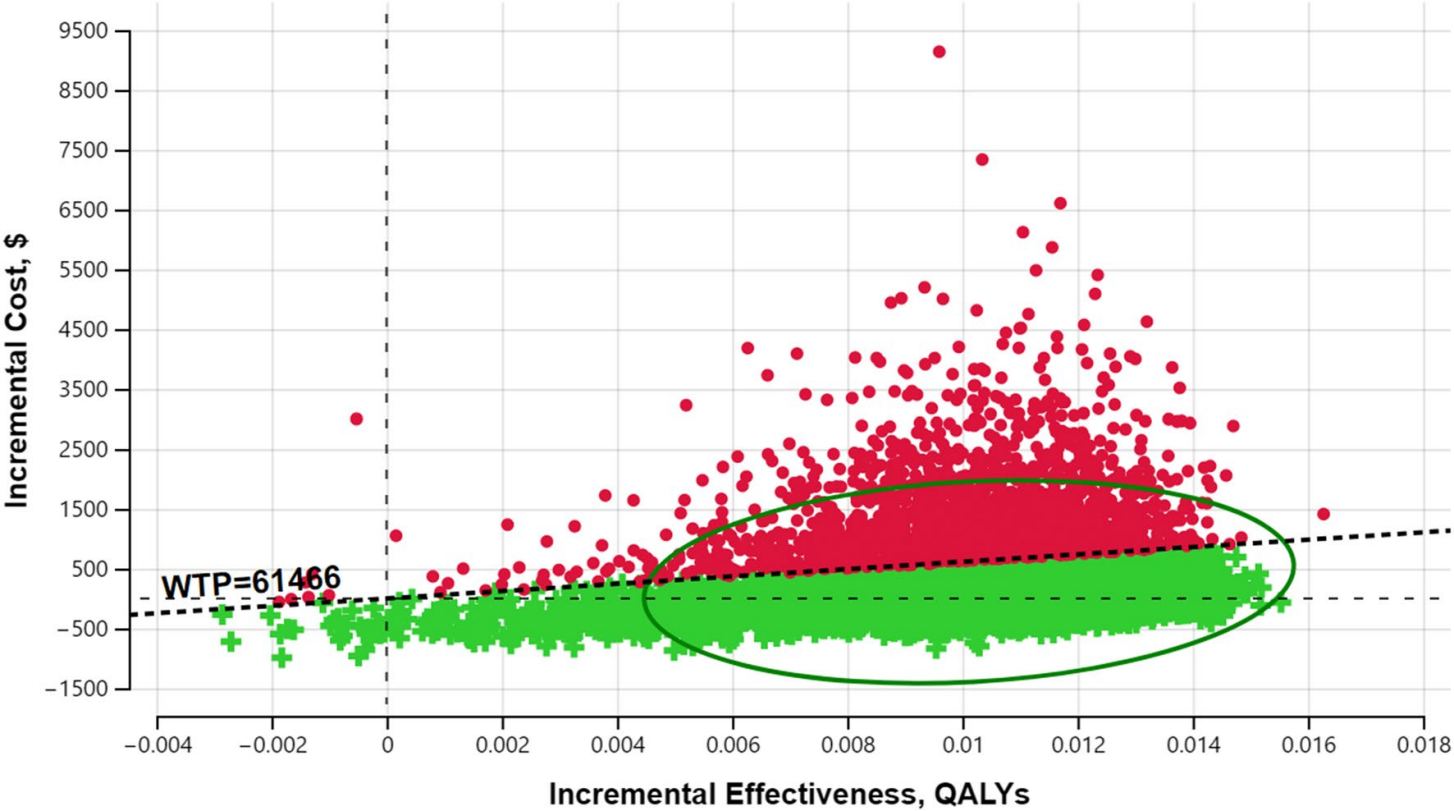
Cost-effectiveness plane for a 1% increase in deceased donor kidney transplantation probability. The cost-effectiveness plane shows the differences in costs and QALYs between the deemed and expressed consent, with the origin depicting the expressed consent. The incremental QALYs (x-axis) represent the differences in expected QALYs between the deemed and the expressed consent. The incremental costs (y-axis) show the differences in expected costs. Each dot in the plane represents one of the 10,000 Monte Carlo simulations from a probabilistic sensitivity analysis

For a minimum of 1% increase in deceased donor KT probability, deemed consent was cost-effective, with an ICUR of $32,629/QALY (95% CI: -64,279 to 232,488) and a probability of cost-effectiveness of 81% if the WTP threshold was $61,466 (Table 3). As the WTP threshold increased, deemed consent's probability of cost-effectiveness also increased based on a PSA (CEAC, Fig. 3). Figure 4, a cost-effectiveness plane, shows the cost and QALY differences from the Monte Carlo Simulations. In the case of a 26% increase, the ICUR was $38,594/QALY (95% CI: -41,022 to 220,930), with an 80% probability that deemed consent was cost-effective if the WTP threshold was $61,466 (Table 3). Again the cost-effectiveness probability increased with the WTP threshold (Additional file 1: Fig. S1).

**Fig. 5:**
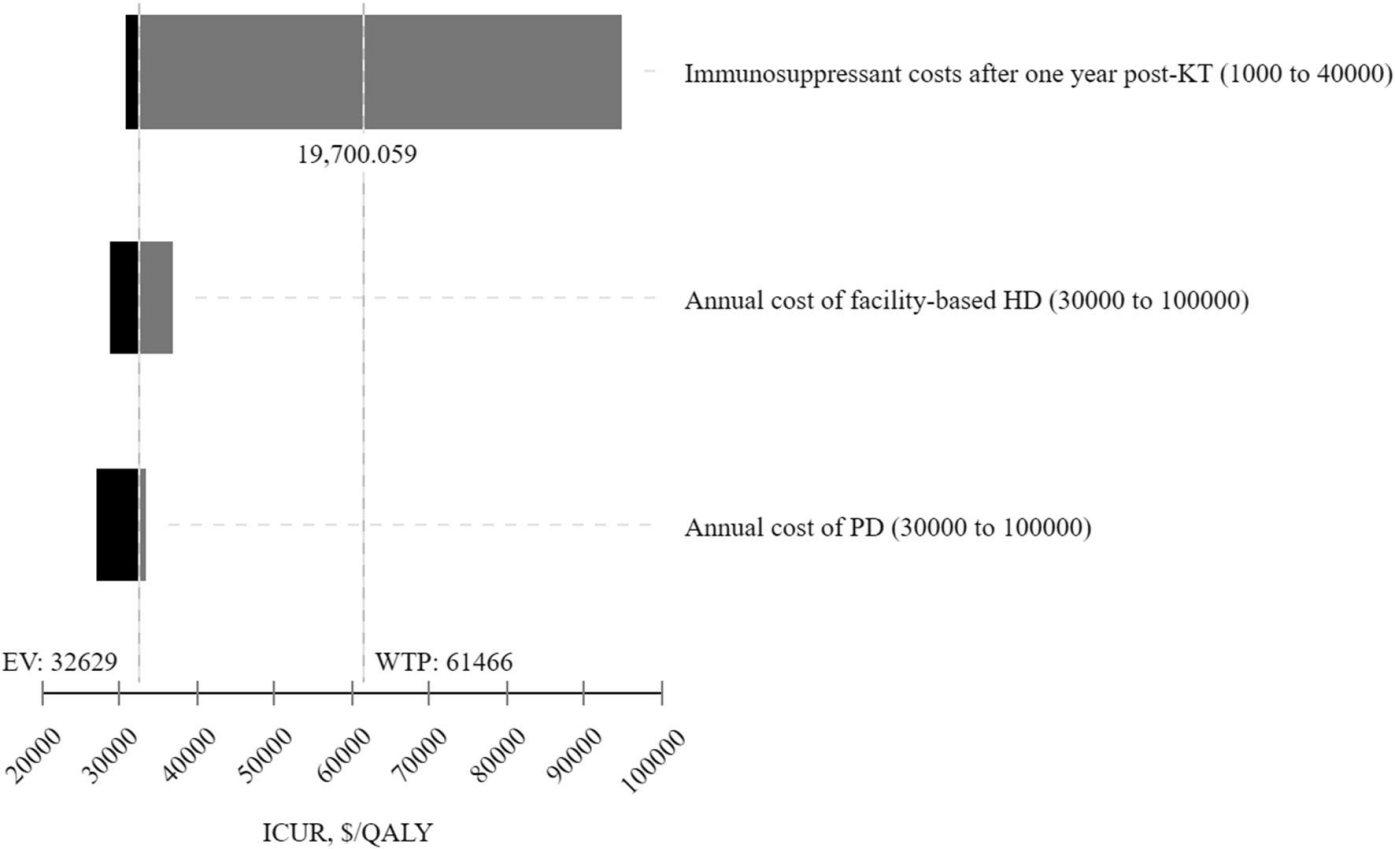
The sensitivity of ICUR to changes in dialysis and maintenance immunosuppressant drug costs for a 1% increase in deceased donor kidney transplantations. *ICUR* incremental cost-utility ratio, *QALY* quality-adjusted life-year, *WTP* willingness to pay, *KT* kidney transplantation, *HD* hemodialysis, *PD* peritoneal dialysis *EV* expected value

Increases in dialysis costs reduced the ICUR, making deemed consent more cost-effective (Fig. 5). As dialysis costs increased, the total costs incurred by the expressed consent arm increased because patients spent relatively more time on dialysis than in the deemed consent arm. As a result, this increase in dialysis costs reduced the cost difference between the two arms. For example, increases in HD costs from $30,000 to $100,000 per year reduced the incremental cost, shrinking the expected lifetime cost difference between the deemed and expressed consent arms of the model, reducing the ICUR (Fig. 5).

On the other hand, increases in maintenance immunosuppressant drug costs increased the ICUR. Assuming a 1% increase in deceased donor KT probability, immunosuppressant drug costs beyond $19,700 per patient per year pushed the ICUR above the WTP threshold, reducing the cost-effectiveness likelihood of deemed consent (Fig. 5). The maintenance immunosuppressant drug cost threshold decreased to $17,000 per patient per year for a 26% increase in deceased donor KT probability (Additional file 1: Fig. S3). If deemed consent contributes to a 100% increase in deceased donor KT probability per year, the immunosuppressant drug cost threshold decreased to $7,200 per patient, given the WTP threshold.

For a 1% increase in deceased donor KT, a corresponding 1% decrease in living donor KT results in higher expected costs and QALYs for deemed consent relative to expressed consent, with a 65% probability that deemed consent is cost-effective. In comparison, a 1.8% decrease in living donor KT, given a 1% increase in deceased donor KT, makes the expected cost associated with deemed consent higher relative to expressed consent with no difference in expected QALYs; hence, expressed consent dominates deemed consent. See Additional file 1: Table S2 for additional results for this scenario. In the case of a 26% increase in deceased donor KT, a corresponding 26% decrease in living donor KT results in deemed consent having higher expected costs and QALYs relative to expressed consent, with a 64% probability that deemed consent is cost-effective. However, for a 43% decrease in living donor KT, assuming a 26% increase in deceased donor KT, deemed consent has higher expected costs with no difference in QALYs; hence, dominated by expressed consent. See Additional file 1: Table S3 for additional results.

## Discussion

Our study was an ex-ante cost-utility analysis of the deemed consent legislation in NS compared to expressed consent, focusing on KT from a payer perspective. We simulated the minimum required increase in deceased donor KT per year for deemed consent to be cost-effective and identified influential parameters. Using a Markov model, we demonstrated that the ICUR increased with the proportion of patients on the waitlist getting a deceased donor KT per year. The cost-effectiveness of the deemed consent legislation for KT required a minimum of a 1% increase in deceased donor KT per year. We also demonstrated that increased costs of maintenance immunosuppressant drugs beyond a threshold negatively impacted the likelihood of cost-effectiveness. That threshold depends on the number of deceased-donor KTs performed in a year and the WTP threshold. In the same vein, increases in dialysis cost enhanced cost-effectiveness likelihood.

The threshold for the annual cost of maintenance immunosuppressant drugs ranged from $7,200 to $19,700. A higher deceased donor KT probability means ESKD patients will spend less time on dialysis and likely enjoy the survival advantage associated with KT. It also means that, in the absence of capacity constraints, more ESKD patients will receive KTs per year, potentially increasing the number of individuals on maintenance immunosuppressant drugs. Also, deemed consent was cost-saving compared to expressed consent in the short run but not in the long run.

We also demonstrated that the cost-effectiveness of deemed consent in NS would depend on whether there is a decrease in living donor KT and how much. Whether deemed consent in NS will negatively impact living donor KT is an empirical question and remains to be seen. Nevertheless, the expectation is that the educational campaigns, as part of the health system transformations, will mitigate potential negative reactions to organ donations in general and, by extension, living organ donations.

KT recipients generally enjoy high quality-adjusted life years compared to those on dialysis and, in some cases, can return to work [42, 43]. In addition, the mean age of KT recipients in Atlantic Canada was 50 years, which falls within the labour force's core age group (25–54 years) [44]. Therefore, it is reasonable to assume that KT recipients can return to the labour force, further contributing to economic growth in the Atlantic region and Canada.

We analyzed a unique policy question. The ex-ante nature of our analysis makes comparing our results to the literature complicated. However, our results match studies on other related policies in the literature [14, 17, 45–47]. Barnieh et al*.* [45], in a Canadian study, examined the cost-effectiveness of paying living kidney donors $10,000 versus the existing donation program. They reported that a transplantation rate increase of 5% would be cost-saving and result in QALY gain than the existing organ donation system. However, they did not include inpatient and other physician-insured service costs before and after KT.

Whiting et al*.* [17] also examined the cost-effectiveness of deceased donor KT compared to dialysis using a Markov model with a 20-year horizon. They evaluated KT within the context of Donor Action, a program designed to increase KT. They reported that the transplantation arm of their model yielded 1.99 QALYs gained and a cost reduction of $104,000 compared to waiting on dialysis. Our study differs from Whiting et al*.* in many ways. First, unlike our study, they compared KT with dialysis. Second, unlike their analysis, our analysis was over a lifetime horizon and included annual inpatient and physician-insured service costs per patient before KT and after KT. Third, we demonstrated the impact time horizon could have on the ICUR by comparing a time horizon of five years to a lifetime.

## Strengths and limitations

The strengths of the present study are that it evaluated a wider range of changes (− 10% to 100%) in deceased-donor KT per year, included other patient-related costs before and after KT and highlighted how dialysis and maintenance immunosuppressant drug costs and decreases in living donor KT could impact cost-effectiveness likelihood. However, the current study has limitations.

The present study was from a payer perspective, not a societal one. Consequently, we did not include patients' out-of-pocket and informal health sector costs, including unpaid caregiver time, transportation, and non-health sector costs such as lost and uncompensated household production [14]. However, a societal perspective would not have changed the conclusions of our current study. We expected patients to spend more time on dialysis and make more trips to the dialysis centre in the expressed consent arm, bearing the transportation, parking and other costs associated with a visit. These patients' out-of-pocket costs will increase the costs associated with expressed relative to deemed consent, thereby improving the likelihood of cost-effectiveness of deemed consent.

Also, the present study was an ex-ante analysis. Therefore, the actual effectiveness of the intervention in terms of the number of deceased donor KTs performed in a year, the number of years patients spend on the waitlist, and the effect on living donor KTs remains to be seen;

An ex-post evaluation of the deemed consent legislation could potentially examine these and their overall impact on the number of KTs in a year.

Also, we did not directly account for the implementation costs and investments in the health system transformation to support the deemed consent model. Directly incorporating the implementation costs would require detailed information on the provincial government's investment spending and for what purpose to determine eligibility. Some of those investments are likely to be sunk costs and hence, no longer relevant for decision making, but that information was not available. However, we indirectly accounted for some of the investments in the analysis. For example, we included a one-time organ procurement cost of $26,943 per patient for KT, the interprovincial billing rate for organ procurement in Canada. We assumed that even NS residents receiving KT in NS incurred this amount. However, in principle, this represents what provinces charge patients from outside their province. We reasoned that the organ procurement cost reflects the transplant coordinators' and specialists' work. By extension, it also reflected the organ donation program investments. In addition, investment in the organ donation program as part of the deemed consent legislation applies to KT and all the other relevant solid organs and tissues, so we would have to split the investment amount among them and hence, unlikely to change the conclusions from the analysis.

## Conclusions

The deemed consent legislation in NS and the concurrent health system transformations are cost-effective compared to expressed consent to the extent that they are anticipated to contribute to more deceased donor KTs in a year. Taken together, deriving the full economic benefits associated with an intervention designed to increase deceased donor KT depends not only on the success of that intervention but also on advances in medical science that improve patient outcomes after KT, including survival and reducing patients' need for maintenance immunosuppressant drugs after KT.

## Supplementary Information

**Additional file 1: Table S1.** Cost-effectiveness analysis results for various changes in deceased donor kidney transplantation probability. **Table S2.** Cost-effectiveness analyses result for various percentage changes in living donor kidney transplantation probability, assuming a 1% increase in deceased donor kidney transplantation probability. **Table S3**. Cost-effectiveness analyses result for various percentage changes in living donor kidney transplantation probability, assuming a 26% increase in deceased donor kidney transplantation probability. **Figure S1.** Cost-effectiveness acceptability curve for a 26% increase in deceased donor kidney transplantation probability. **Figure S2.** Cost-effectiveness plane for a 26% increase in deceased donor kidney transplantation probability. **Figure S3.** The sensitivity of ICUR to changes in dialysis and maintenance immunosuppressant drug costs for a 26% increase in deceased donor KT probability. ICUR, incremental cost-utility ratio; QALY, quality-adjusted life-year; WTP, willingness to pay; KT, kidney transplantation; HD, hemodialysis; PD, peritoneal dialysis; EV expected value.

## Data Availability

All the data used to parameterize the model are included in this published article.
