# Supplementary material for: An ex-ante cost-utility analysis of the deemed consent legislation compared to expressed consent for kidney transplantations in Nova Scotia
Source: Cost Eff Resour Alloc. 2022 Oct 6;20:55. doi: 10.1186/s12962-022-00390-z (PMC9535887; doi:10.1186/s12962-022-00390-z)
Supplement: Supplementary file 1 — Additional file 1: Table S1. Cost-effectiveness analysis results for various changes in deceased donor kidney transplantation probability. Table S2. Cost-effectiveness analyses result for various percentage changes in living donor kidney transplantation probability, assuming a 1% increase in deceased donor kidney transplantation probability. Table S3. Cost-effectiveness analyses result for various percentage changes in living donor kidney transplantation probability, assuming a 26% increase in deceased donor kidney transplantation probability. Figure S1. Cost-effectiveness acceptability curve for a 26% increase in deceased donor kidney transplantation probability. Figure S2. Cost-effectiveness plane for a 26% increase in deceased donor kidney transplantation probability. Figure S3. The sensitivity of ICUR to changes in dialysis and maintenance immunosuppressant drug costs for a 26% increase in deceased donor KT probability. ICUR, incremental cost-utility ratio; QALY, quality-adjusted life-year; WTP, willingness to pay; KT, kidney transplantation; HD, hemodialysis; PD, peritoneal dialysis; EV expected value. [file 12962_2022_390_MOESM1_ESM.docx]

**Supplementary Tables and Figures**

Table S1. Cost-effectiveness analysis results for various changes in deceased donor kidney transplantation probability

| Percent change | Strategy | Cost | ∆ Cost | QALYs | ∆QALYs | ICUR ($/QALY) |
| --- | --- | --- | --- | --- | --- | --- |
| -10% | Deemed consent | $305,780 | $2,386 | 3.76 | 0.10 | 24,785 |
|  | Expressed consent | $308,167 |  | 3.85 |  |  |
| -5% | Deemed consent | $306,908 | $1,259 | 3.81 | 0.05 | 27,955 |
|  | Expressed consent | $308,167 |  | 3.85 |  |  |
| 1% | Expressed consent | $308,167 | $349 | 3.85 | 0.01 | 32,629 |
|  | Deemed consent | $308,516 |  | 3.86 |  |  |
| 6% | Expressed consent | $308,167 | $2,407 | 3.85 | 0.07 | 34,021 |
|  | Deemed consent | $310,573 |  | 3.92 |  |  |
| 12% | Expressed consent | $308,167 | $4,695 | 3.85 | 0.13 | 35,332 |
|  | Deemed consent | $312,861 |  | 3.99 |  |  |
| 18% | Expressed consent | $308,167 | $7,227 | 3.85 | 0.20 | 36,625 |
|  | Deemed consent | $315,393 |  | 4.05 |  |  |
| 23% | Expressed consent | $308,167 | $10,019 | 3.85 | 0.26 | 37,904 |
|  | Deemed consent | $318,186 |  | 4.12 |  |  |
| 29% | Expressed consent | $308,167 | $13,087 | 3.85 | 0.33 | 39,165 |
|  | Deemed consent | $321,254 |  | 4.19 |  |  |
| 40% | Expressed consent | $308,167 | $20,128 | 3.85 | 0.48 | 41,634 |
|  | Deemed consent | $328,295 |  | 4.34 |  |  |
| 45% | Expressed consent | $308,167 | $24,345 | 3.85 | 0.57 | 43,019 |
|  | Deemed consent | $332,512 |  | 4.42 |  |  |
| 56% | Expressed consent | $308,167 | $34,446 | 3.85 | 0.75 | 45,918 |
|  | Deemed consent | $342,613 |  | 4.60 |  |  |
| 62% | Expressed consent | $308,167 | $40,314 | 3.85 | 0.85 | 47,317 |
|  | Deemed consent | $348,481 |  | 4.70 |  |  |
| 73% | Expressed consent | $308,167 | $53,584 | 3.85 | 1.07 | 49,873 |
|  | Deemed consent | $361,751 |  | 4.93 |  |  |
| 78% | Expressed consent | $308,167 | $61,076 | 3.85 | 1.20 | 51,056 |
|  | Deemed consent | $369,243 |  | 5.05 |  |  |
| 84% | Expressed consent | $308,167 | $69,218 | 3.85 | 1.33 | 52,192 |
|  | Deemed consent | $377,384 |  | 5.18 |  |  |
| 89% | Expressed consent | $308,167 | $78,053 | 3.85 | 1.46 | 53,280 |
|  | Deemed consent | $386,219 |  | 5.32 |  |  |
| 95% | Expressed consent | $308,167 | $87,639 | 3.85 | 1.61 | 54,324 |
|  | Deemed consent | $395,806 |  | 5.47 |  |  |
| 100% | Expressed consent | $308,167 | $98,043 | 3.85 | 1.77 | 55,328 |
|  | Deemed consent | $406,210 |  | 5.62 |  |  |

Table S2. Cost-effectiveness analyses result for various percentage changes in living donor kidney transplantation probability, assuming a 1% increase in deceased donor kidney transplantation probability

| Percent change | Strategy | Cost | ∆ Cost | QALYs | ∆QALYs | ICUR ($/QALY) |
| --- | --- | --- | --- | --- | --- | --- |
| -100% | Expressed consent | $308,167 | $9,071 | 3.85 | -0.63 | Deemed consent dominated |
|  | Deemed consent | $317,238 |  | 3.22 |  |  |
| -95% | Expressed consent | $308,167 | $8,227 | 3.85 | -0.59 | Deemed consent dominated |
|  | Deemed consent | $316,393 |  | 3.26 |  |  |
| -90% | Expressed consent | $308,167 | $7,418 | 3.85 | -0.56 | Deemed consent dominated |
|  | Deemed consent | $315,584 |  | 3.29 |  |  |
| -80% | Expressed consent | $308,167 | $5,903 | 3.85 | -0.49 | Deemed consent dominated |
|  | Deemed consent | $314,070 |  | 3.36 |  |  |
| -75% | Expressed consent | $308,167 | $5,197 | 3.85 | -0.46 | Deemed consent dominated |
|  | Deemed consent | $313,364 |  | 3.40 |  |  |
| -70% | Expressed consent | $308,167 | $4,525 | 3.85 | -0.42 | Deemed consent dominated |
|  | Deemed consent | $312,692 |  | 3.43 |  |  |
| -65% | Expressed consent | $308,167 | $3,886 | 3.85 | -0.39 | Deemed consent dominated |
|  | Deemed consent | $312,052 |  | 3.46 |  |  |
| -50% | Expressed consent | $308,167 | $2,165 | 3.85 | -0.30 | Deemed consent dominated |
|  | Deemed consent | $310,332 |  | 3.56 |  |  |
| -45% | Expressed consent | $308,167 | $1,656 | 3.85 | -0.27 | Deemed consent dominated |
|  | Deemed consent | $309,823 |  | 3.59 |  |  |
| -40% | Expressed consent | $308,167 | $1,218 | 3.85 | -0.24 | Deemed consent dominated |
|  | Deemed consent | $309,384 |  | 3.62 |  |  |
| -35% | Expressed consent | $308,167 | $842 | 3.85 | -0.20 | Deemed consent dominated |
|  | Deemed consent | $309,009 |  | 3.65 |  |  |
| -30% | Expressed consent | $308,167 | $499 | 3.85 | -0.18 | Deemed consent dominated |
|  | Deemed consent | $308,666 |  | 3.68 |  |  |
| -25% | Expressed consent | $308,167 | $223 | 3.85 | -0.15 | Deemed consent dominated |
|  | Deemed consent | $308,390 |  | 3.71 |  |  |
| -20% | Expressed consent | $308,167 | $80 | 3.85 | -0.11 | Deemed consent dominated |
|  | Deemed consent | $308,247 |  | 3.74 |  |  |
| -15% | Expressed consent | $308,167 | $24 | 3.85 | -0.08 | Deemed consent dominated |
|  | Deemed consent | $308,191 |  | 3.77 |  |  |
| -10% | Expressed consent | $308,167 | $2 | 3.85 | -0.05 | Deemed consent dominated |
|  | Deemed consent | $308,169 |  | 3.80 |  |  |
| -5% | Expressed consent | $308,167 | $129 | 3.85 | -0.02 | Deemed consent dominated |
|  | Deemed consent | $308,296 |  | 3.83 |  |  |
| 0% | Expressed consent | $308,167 | $349 | 3.85 | 0.01 | 32,629 |
|  | Deemed consent | $308,516 |  | 3.86 |  |  |

Table S3. Cost-effectiveness analyses result for various percentage changes in living donor kidney transplantation probability, assuming a 26% increase in deceased donor kidney transplantation probability

| Percent change | Strategy | Cost | ∆ Cost | QALYs | ∆QALYs | ICUR ($/QALY) |
| --- | --- | --- | --- | --- | --- | --- |
| -100% | Expressed consent | $308,167 | $8,126 | 3.85 | -0.39 | Deemed consent dominated |
|  | Deemed consent | $316,292 |  | 3.46 |  |  |
| -95% | Expressed consent | $308,167 | $7,641 | 3.85 | -0.36 | Deemed consent dominated |
|  | Deemed consent | $315,808 |  | 3.50 |  |  |
| -90% | Expressed consent | $308,167 | $7,192 | 3.85 | -0.32 | Deemed consent dominated |
|  | Deemed consent | $315,359 |  | 3.53 |  |  |
| -80% | Expressed consent | $308,167 | $6,421 | 3.85 | -0.26 | Deemed consent dominated |
|  | Deemed consent | $314,587 |  | 3.60 |  |  |
| -75% | Expressed consent | $308,167 | $6,178 | 3.85 | -0.22 | Deemed consent dominated |
|  | Deemed consent | $314,345 |  | 3.63 |  |  |
| -70% | Expressed consent | $308,167 | $6,078 | 3.85 | -0.19 | Deemed consent dominated |
|  | Deemed consent | $314,244 |  | 3.66 |  |  |
| -65% | Expressed consent | $308,167 | $6,013 | 3.85 | -0.15 | Deemed consent dominated |
|  | Deemed consent | $314,180 |  | 3.70 |  |  |
| -50% | Expressed consent | $308,167 | $6,302 | 3.85 | -0.05 | Deemed consent dominated |
|  | Deemed consent | $314,468 |  | 3.80 |  |  |
| -45% | Expressed consent | $308,167 | $6,651 | 3.85 | -0.02 | Deemed consent dominated |
|  | Deemed consent | $314,818 |  | 3.84 |  |  |
| -40% | Expressed consent | $308,167 | $7,047 | 3.85 | 0.02 | 362,812 |
|  | Deemed consent | $315,214 |  | 3.87 |  |  |
| -35% | Expressed consent | $308,167 | $7,482 | 3.85 | 0.05 | 136,354 |
|  | Deemed consent | $315,649 |  | 3.91 |  |  |
| -30% | Expressed consent | $308,167 | $7,957 | 3.85 | 0.09 | 88,172 |
|  | Deemed consent | $316,123 |  | 3.94 |  |  |
| -25% | Expressed consent | $308,167 | $8,471 | 3.85 | 0.13 | 67,472 |
|  | Deemed consent | $316,638 |  | 3.98 |  |  |
| -20% | Expressed consent | $308,167 | $9,026 | 3.85 | 0.16 | 56,122 |
|  | Deemed consent | $317,192 |  | 4.01 |  |  |
| -15% | Expressed consent | $308,167 | $9,621 | 3.85 | 0.20 | 49,065 |
|  | Deemed consent | $317,787 |  | 4.05 |  |  |
| -10% | Expressed consent | $308,167 | $10,258 | 3.85 | 0.23 | 44,337 |
|  | Deemed consent | $318,425 |  | 4.08 |  |  |
| -5% | Expressed consent | $308,167 | $10,937 | 3.85 | 0.27 | 41,011 |
|  | Deemed consent | $319,103 |  | 4.12 |  |  |
| 0% | Expressed consent | $308,167 | $11,657 | 3.85 | 0.30 | 38,594 |
|  | Deemed consent | $319,824 |  | 4.15 |  |  |


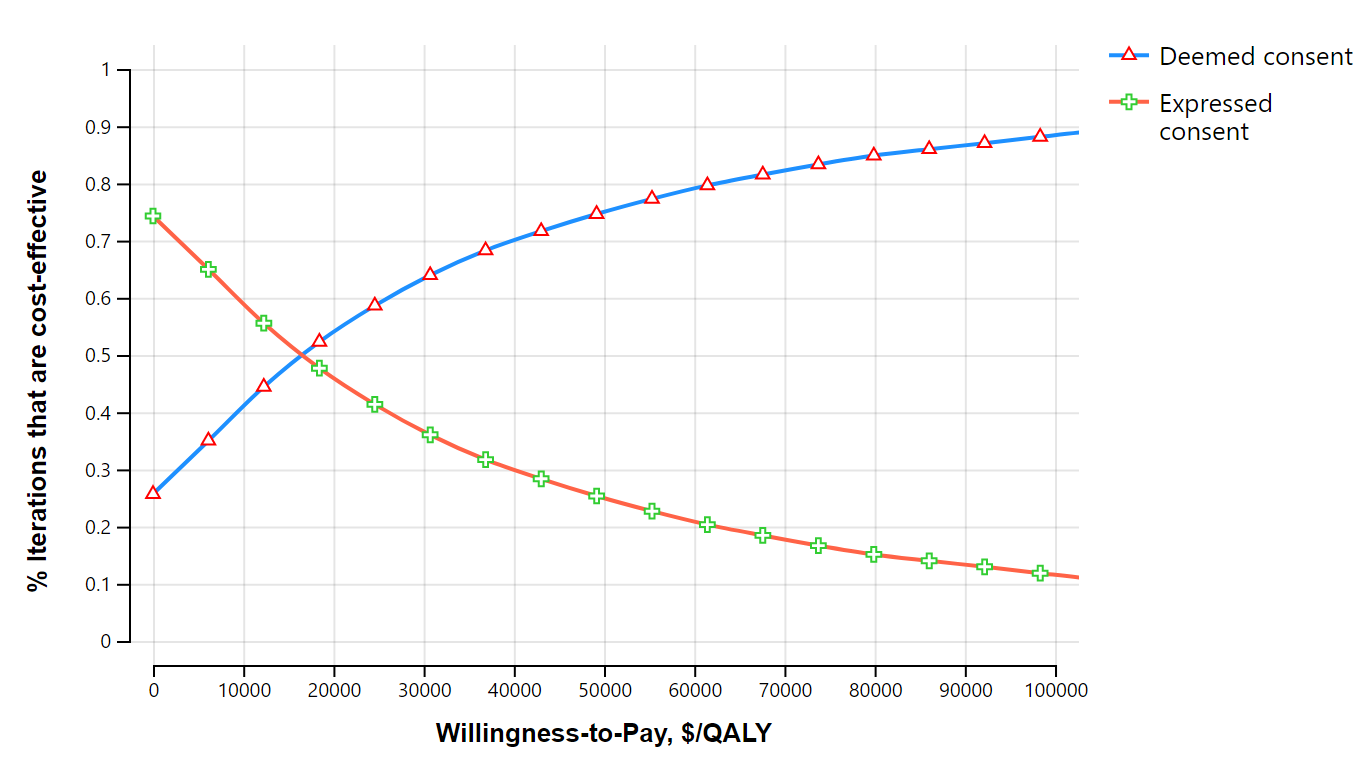


Figure S1. Cost-effectiveness acceptability curve for a 26% increase in deceased donor kidney transplantation probability.


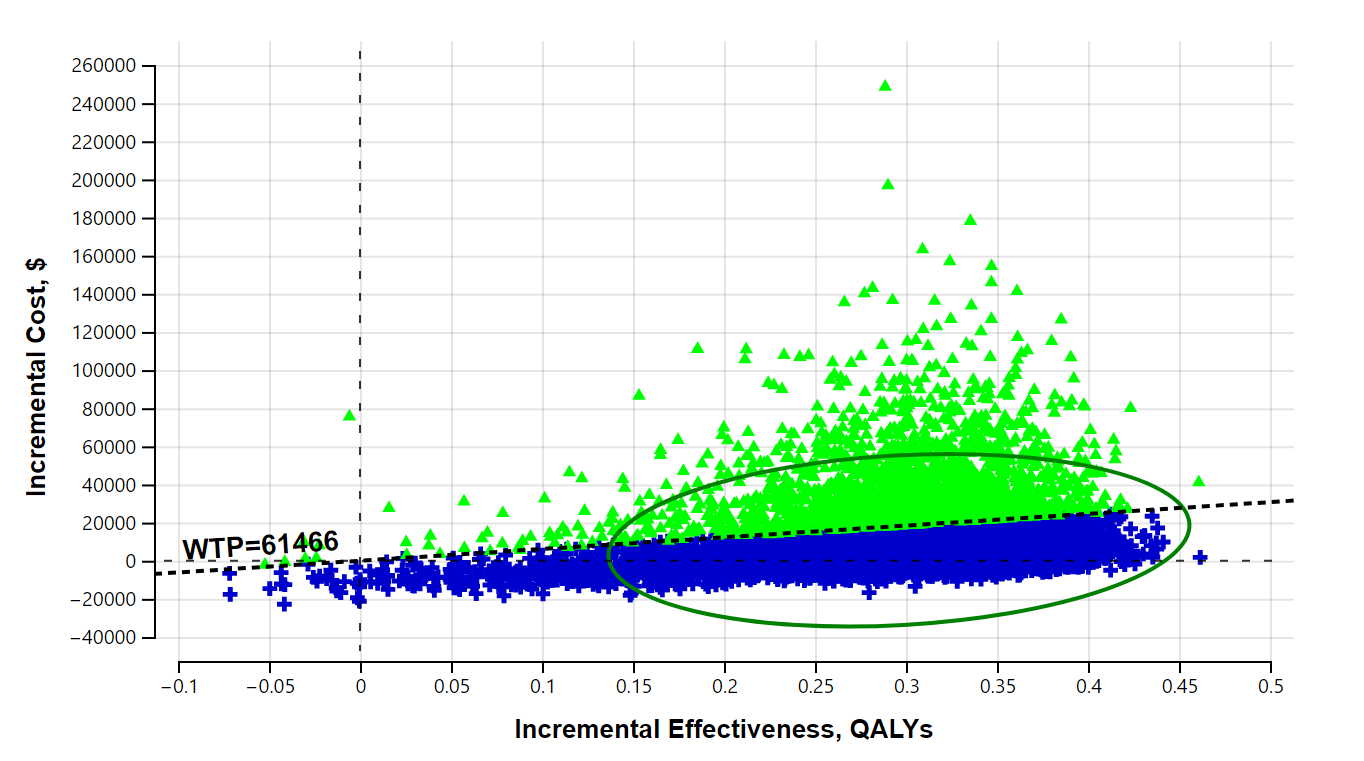


Figure S2. Cost-effectiveness plane for a 26% increase in deceased donor kidney transplantation probability.


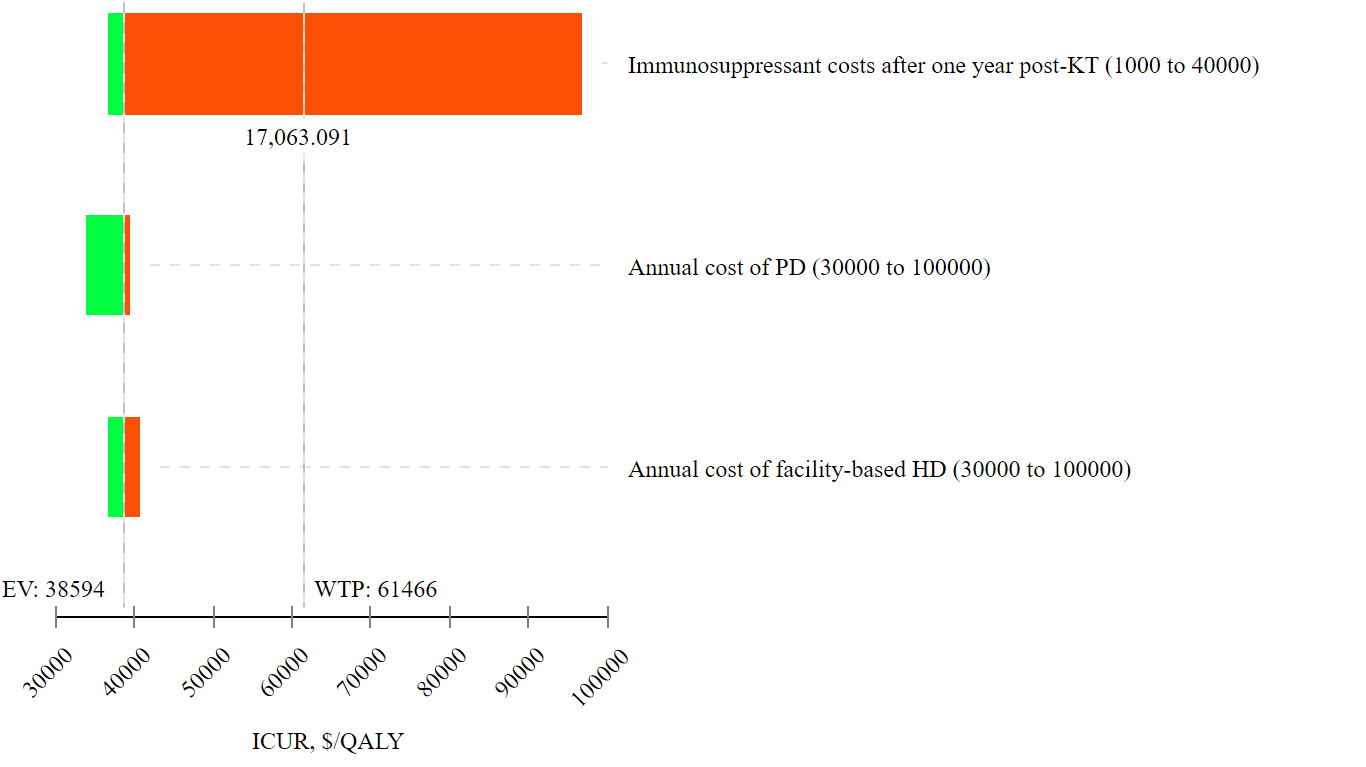


Figure S3. The sensitivity of ICUR to changes in dialysis and maintenance immunosuppressant drug costs for a 26% increase in deceased donor KT probability. ICUR, incremental cost-utility ratio; QALY, quality-adjusted life-year; WTP, willingness to pay; KT, kidney transplantation; HD, hemodialysis; PD, peritoneal dialysis; EV expected value.
